# Disrupted Membrane Structure and Intracellular Ca^2+^ Signaling in Adult Skeletal Muscle with Acute Knockdown of Bin1

**DOI:** 10.1371/journal.pone.0025740

**Published:** 2011-09-30

**Authors:** Andoria Tjondrokoesoemo, Ki Ho Park, Christopher Ferrante, Shinji Komazaki, Sebastian Lesniak, Marco Brotto, Jae-Kyun Ko, Jingsong Zhou, Noah Weisleder, Jianjie Ma

**Affiliations:** 1 Department of Physiology and Biophysics, Robert Wood Johnson Medical School, University of Medicine and Dentistry of New Jersey (UMDNJ), Piscataway, New Jersey, United States of America; 2 Department of Anatomy, Saitama Medical School, Saitama, Japan; 3 Muscle Biology Research Group, University of Missouri, Kansas City, Missouri, United States of America; 4 Department of Molecular Biophysics and Physiology, Rush University, Chicago, Illinois, United States of America; Brigham & Women's Hospital - Harvard Medical School, United States of America

## Abstract

Efficient intracellular Ca^2+^ ([Ca^2+^]i) homeostasis in skeletal muscle requires intact triad junctional complexes comprised of t-tubule invaginations of plasma membrane and terminal cisternae of sarcoplasmic reticulum. Bin1 consists of a specialized BAR domain that is associated with t-tubule development in skeletal muscle and involved in tethering the dihydropyridine receptors (DHPR) to the t-tubule. Here, we show that Bin1 is important for Ca^2+^ homeostasis in adult skeletal muscle. Since systemic ablation of Bin1 in mice results in postnatal lethality, *in vivo* electroporation mediated transfection method was used to deliver RFP-tagged plasmid that produced short –hairpin (sh)RNA targeting Bin1 (shRNA-Bin1) to study the effect of Bin1 knockdown in adult mouse FDB skeletal muscle. Upon confirming the reduction of endogenous Bin1 expression, we showed that shRNA-Bin1 muscle displayed swollen t-tubule structures, indicating that Bin1 is required for the maintenance of intact membrane structure in adult skeletal muscle. Reduced Bin1 expression led to disruption of t-tubule structure that was linked with alterations to intracellular Ca^2+^ release. Voltage-induced Ca^2+^ released in isolated single muscle fibers of shRNA-Bin1 showed that both the mean amplitude of Ca^2+^ current and SR Ca^2+^ transient were reduced when compared to the shRNA-control, indicating compromised coupling between DHPR and ryanodine receptor 1. The mean frequency of osmotic stress induced Ca^2+^ sparks was reduced in shRNA-Bin1, indicating compromised DHPR activation. ShRNA-Bin1 fibers also displayed reduced Ca^2+^ sparks' amplitude that was attributed to decreased total Ca^2+^ stores in the shRNA-Bin1 fibers. Human mutation of Bin1 is associated with centronuclear myopathy and SH3 domain of Bin1 is important for sarcomeric protein organization in skeletal muscle. Our study showing the importance of Bin1 in the maintenance of intact t-tubule structure and ([Ca^2+^]i) homeostasis in adult skeletal muscle could provide mechanistic insight on the potential role of Bin1 in skeletal muscle contractility and pathology of myopathy.

## Introduction

Bin1 is a member of Amphiphysin family of proteins that contains a canonical NH_2_-terminal BAR (Bin/Amphiphysin/Rvs) protein domain which induces membrane bending, a Src homology 3 (SH3) domain at the carboxy-terminus and a variable region at the center of the protein [1], [2]. Although Bin1 was initially characterized as a tumor suppressor protein through its interaction with Myc protein, Bin1 is also highly expressed in striated muscle with primary localization along the transverse (t)-tubule membrane [3], [4], [5], [6]. Exon 11 of Bin1 consists of PI(4,5)P_2_ binding domain and is thought to be essential for muscle cell fusion and differentiation as it has been shown to be required for the C2C12 myotube formation [4], [7].

Disruption of Bin1 in *drosophila melanogaster* results in viable, flightless flies with disrupted t-tubule structure [8]. Knock-in mice overexpressing SH3 domain of Bin1 suggests that the SH3 domain is associated with actin, myosin filaments and pro-myogenic kinase CDK 5 proteins that are important for sarcomeric assembly and organization [9]. Furthermore, studies in cultured cardiomyocytes suggested that Bin1 is important for proper localization of DHPR (or Ca_V_ 1.2), a family of L-type Ca^2+^ channels, to the t-tubule membrane [10]. While these studies indicate a role for Bin1 in establishing the muscle cell ultrastructure and excitation-contraction (EC) coupling, limited progress has been made in the study of the role of Bin1 in Ca^2+^ homeostasis in adult skeletal muscle due to the lethality associated with systemic ablation of Bin1 in mice. *Bin1(−/−)* mice die shortly after birth due to hypertrophic dilated cardiomyopathy without apparent defects in vesicle trafficking, suggesting one role for Bin1 during cardiac development [11].

Since Bin1 may have distinct functions during development and in adult tissues, the objective of this study was to characterize the specific function of Bin1 in maintenance of t-tubule structure and Ca^2+^ regulation in adult skeletal muscle. We examined the function of Bin1 by applying short hairpin (sh) RNA to silence Bin1 expression in adult flexor digitorum brevis (FDB) skeletal muscle. Using *in vivo* electroporation mediated-transfection [12], [13], [14], [15], [16] of plasmids that produce shRNA against Bin1, we found that knockdown of Bin1in adult mice disrupted the t-tubule structure, suggesting that Bin1 is also required also for the maintenance of intact membrane structure in adult skeletal muscle as well as during development.

Intact triad junction structure in skeletal muscle is required for effective conformational coupling of (DHPR), a voltage sensor and L-type gated Ca^2+^ channel at the t-tubule membrane, to ryanodine receptor 1 (RyR1), a Ca^2+^ release channel at the sarcoplasmic reticulum (SR) [17], [18]. Since previous studies showed that knockdown of Bin in the heart led to a delayed Ca^2+^ transient [10], it is possible that the alteration of triad junction structure following the knockdown of Bin1 in the adult skeletal muscle could affect intracellular Ca^2+^ release. To test this possibility, we measured voltage induced Ca^2+^ release (VICR) in these fibers. The voltage-current relationship (IV plot) showed that mean amplitude of inward Ca^2+^ current was altered in the shRNA-Bin1 fibers. Additionally, the mean amplitude of Ca^2+^ transients were reduced, further suggesting ineffective conformational coupling between DHPR and RyR. While VICR is an indicator of global Ca^2+^ release, Ca^2+^ sparks measure the opening of clustered and localized RyRs. Earlier studies revealed that dystrophic muscle or aged muscle display altered Ca^2+^ sparks activity when compared to young and healthy muscle, suggesting the possible physiological significance of the osmotic stress induced Ca^2+^ sparks [19], [20], [21]. In the shRNA-Bin1 fibers, we observed reduced Ca^2+^ spark frequency and amplitude. The reduction of Ca^2+^ sparks amplitude could be partially attributed to a decreased Ca^2+^ store within the SR, suggesting that alteration in membrane structure could directly modulate Ca^2+^ storage, which would lead to compromised SR Ca^2+^ release.

## Results

### shRNA-mediated knockdown of Bin1 in adult skeletal muscle

**Figure 1:**
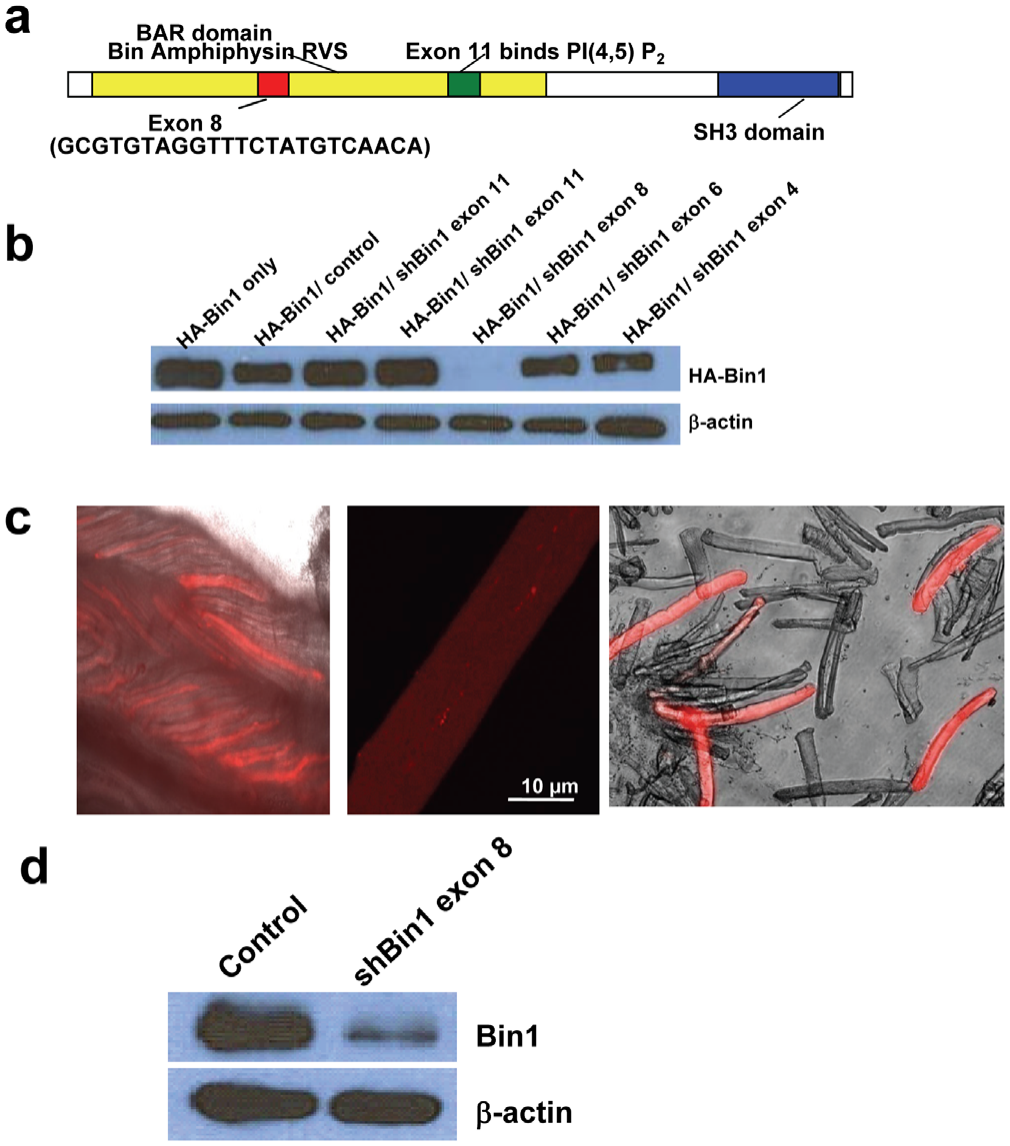
shRNA-mediated knockdown of Bin1 in adult skeletal muscle. (a) Protein domains of Bin1. The listed nucleotide sequence in exon 8 represents the shRNA against Bin1 (b) Western blots of whole cell extracts from HEK293 co-transfected with a murine Bin1 cDNA plasmid and various shRNA constructs against Bin1. This assay allowed screening for the most effective shRNA at knocking down Bin1 expression. (c) High efficiency gene delivery at 14 days-post electroporation was achieved as shown by the high expression of RFP signals on the flexor digitorum brevis (FDB) muscle bundles that were electroporated with either shRNA-Bin1 or shRNA-control plasmid (*left*). RFP signal was used to identify individually transfected single muscle fiber *(middle*, *right*). (d) Western blot was performed on the trimmed FDB muscle bundle that was transfected with either shRNA-control or shRNA Bin1 to confirm the potency of shRNA-Bin1 in knocking down endogenous Bin1 expression in adult skeletal muscle (n = 4 mice).

Four different shRNA oligonucleotides targeting specific exons of the skeletal muscle of mouse Bin1 mRNA were designed to allow RNAi mediated knockdown of gene expression (Fig. 1a). These sequences were ligated into an expression vector containing red fluorescence protein (RFP) cassette under control of separate promoter, allowing for identification of transfected cells. Another plasmid containing a shRNA sequence against the luciferase cDNA [17] served as a shRNA-control for all of the experiments detailed here. Heterologous expression into HEK293 cells showed that shRNA designed against exon 8 of Bin1 was the most effective in knocking down Bin1 protein expression (Fig. 1b).

shRNA-Bin1 against exon 8 or shRNA-control plasmids were injected into separate hindlimb paws of viable 3-6 months old mice, and then electroporated following previously established protocols [12], [13], [14], [15], [16]. Electroporated FDB muscle bundles were dissected at 14 days post-electroporation to ensure proper recovery of muscle function and structure and avoid effects of tissue regeneration [12], [13]. RFP labeling was used to identify transfected fibers as the transfection efficiency can vary as a result of the electroporation method. As illustrated in Fig. 1c, a high percentage of FDB muscle bundle exhibited RFP labeling indicating that effective gene delivery was achieved. Western blots performed on shRNA-Bin FDB muscle bundles showed significant knockdown of endogenous Bin1 expression in the FDB (Fig. 1d).

### Altered t-tubule structure and increased membrane fragility in adult muscle following knockdown of Bin1

**Figure 2:**
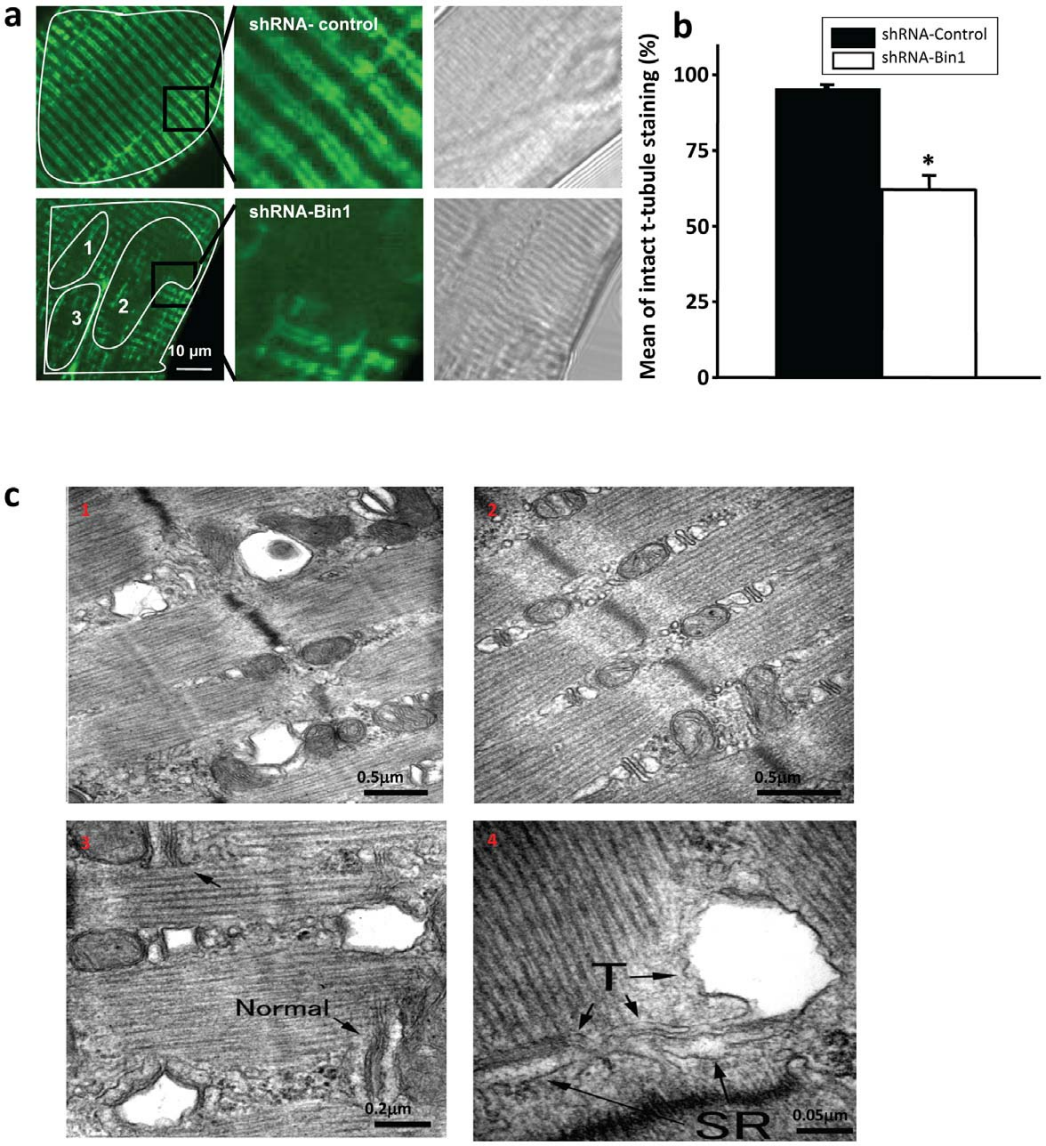
Disrupted t-tubule structure in adult shRNA-Bin1 muscle. (a,*left*) Individually isolated FDB muscle fiber that was electroporated with either shRNA-control or shRNA-Bin1 plasmid was stained with 100 nM DiOC5 that labeled intact t-tubule membrane structure. shRNA-Bin1 fibers exhibited loss or diffused of t-tubule staining in shRNA-Bin1 muscle fibers (n = 13), that otherwise were not observed in the shRNA-control fibers (n = 13). Enlarged images showed that bifurcated t-tubule doublets characteristics were missing in the shRNA-Bin1 fibers (*middle*). Brightfield images *(right*) of cross-striated pattern in both shRNA-control and shRNA-Bin1 indicated healthy muscle fibers were chosen for DiOC5 staining. Several regions of interests (ROI) were drawn on the areas of the fibers that displayed blank DiOC5 staining (numerically labeled small ROIs) and then the total affected area in each fiber was divided by the total surface area of the fiber (large ROI drawn covering the surface area of the fiber). Black box represents the enlarged area showing the bifurcated t-tubule staining. (b)Quantification of missing/diffused staining observed in shRNA-Bin1 (blank square) when compared to shRNA-control (filled square) fibers. (c) EM analysis performed on the trimmed electroporated FDB muscle bundle showed that 25–30% of cells in the FDB muscle bundle transfected with shRNA-Bin1 displayed vacuolation/swollen t-tubule structure (c_1_), whereas the remainder ∼70% exhibited normal t-tubule structure as seen in shRNA-control FDB muscle bundle (c_2_). (c_3,4_) Enlargement of the cells showing swollen t-tubule structure from the shRNA-Bin FDB muscle bundle. Arrows in (c_3_) indicate normal t-tubules while in (c_4_) the arrows indicate enlarged/vacuolated t-tubule structures in shRNA-Bin1 fibers. n = 56 for shRNA-control cells, and n = 63 for shRNA-Bin1 cells.

Considering previous reports on the role of Bin1 in t-tubule formation during muscle development, we tested if reduced Bin1 expression could alter t-tubule structure in adult skeletal muscle. DiOC5 fluorescent dye was used to visualize t-tubules of individual transfected muscle fibers to assess the membrane structure of these fibers. Bifurcated t-tubule doublets were observed in the shRNA-control fibers (Fig. 2a top). shRNA-Bin1 fibers displayed diffused or “blank” t-tubule staining (Fig. 2a bottom), suggesting that any t-tubules in that area were no longer contiguous with the extracellular space. Quantification of DiOC5 staining from individual muscle fibers showed that in average 35%–40% of shRNA-Bin1 fiber surface area in confocal microscopy sections exhibited blank t-tubule staining, whereas shRNA-control fibers exhibited less than 4% of blank t-tubule staining on average (Fig. 2b).

To further analyze the disrupted t-tubule structure in the shRNA-Bin1 muscle fiber we examined the ultrastructure of transfected FDB muscle bundle using electron microscopy (EM). We found that ∼30% of the FDB muscle bundle (19 of 63 cells) transfected with shRNA-Bin1 showed swollen t-tubules structures (Fig. 2c_1_), which altered the integrity of triad junction in affected portions of the muscle fiber (Fig. 2c_3,4_). Conversely, all FDB muscle bundles that were transfected with shRNA-control showed normal triad junction (Fig. 2c_2_). Both the DiOC5 staining and EM data indicate that Bin1 is involved in the maintenance of t-tubule structure in adult muscle.

### Inefficient voltage induced Ca ^2+^ release (VICR) in shRNA-Bin1 muscle

**Figure 3:**
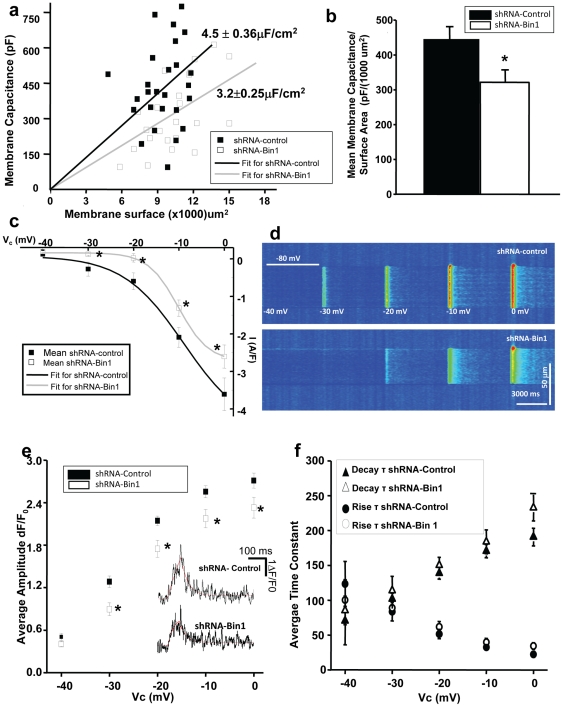
Inefficient voltage induced Ca^2+^ release (VICR) in shRNA-Bin1 muscle. (a) Measured membrane capacitance as a function of the estimated surface membrane area of the silicone-free surface area the fiber. The capacitance was measured by integrating the current transient obtained from 10 mV voltage pulse. Each symbol in the graph corresponds to different fibers, with filled box as shRNA-control and empty box as shRNA-Bin1. Data points, obtained in the same fiber, were fitted to a straight line to zero following the method described by Jacquemond, et al [23] with black line represents the shRNA-control and gray line represents shRNA-Bin1 fibers. (b) Quantification of reduced mean membrane capacitance/surface area in the shRNA-Bin1 (blank square) when compared to shRNA-control fibers (filled square). (c) The voltage-current relationship (IV) from depolarizing pulses of −40 mV to 0 mV was fitted following sigmoidal fit with black line representing shRNA-control and gray line representing shRNA-Bin1. Altered mean current amplitude in the shRNA-Bin1 (blank square) when compared to shRNA-control fibers (filled square). (d) Representative Fluo-4 fluorescence trace during different depolarizing pulses ranging from −40 mV to 0 mV for 200 ms with 10 mV increment from an initial holding voltage of −80 mV. Between pulses the membrane was returned to −80 mV as indicated by the white horizontal bar. The difference of Ca^2+^ transient amplitude after depolarizing stimuli was evident even at −30 mV stimulation. (e) shRNA-Bin1 (n = 26 fibers, blank square) continuously displayed reduced mean amplitude Ca^2+^ release when compared to shRNA-control (n = 22 fibers, filled square) at different depolarizing pulses ranging from -40 to 0 mV. Representative trace of Ca^2+^ release amplitude (dF/F_0_) and kinetic (D_50_) (*inset*). (f) Mean rise (circle) and decay (triangle) τ for both shRNA-control and shRNA-Bin1 fibers were fitted exponentially. No significant differences, however, were observed in these two groups.

Proper positioning of t-tubules and SR is essential for maintenance of Ca^2+^ homeostasis in striated muscle. To determine whether knocking down Bin1 could alter the conformational coupling between DHPR to RyR, we measured VICR in isolated muscle fibers using a silicone-grease embedding method [22], [23], [24]. The mean slope of capacitance measurement against surface area of fibers evoked during 10 mV hyperpolarizing command was lower in the shRNA-Bin1 fibers (3.2±0.25 µF/cm2) when compared to the shRNA-control fibers (4.5 ± 0.36 µF/cm2) (Fig. 3a, b). This suggests that shRNA-Bin1 fibers could have less total membrane surface than control fibers. Such a situation would be expected considering that disrupted t-tubule structure observed in the DiOC5 staining and EM studies (Fig. 2) would result in less total surface of the sarcolemmal that could be depolarized. Isolated fibers were stimulated with depolarizing pulses ranging from −40 mV to 0 mV for 200 ms each with 10 mV increments from a holding potential of −80 mV (Fig. 3c,d). The voltage-current relationship (IV) plot of both shRNA-control and shRNA-Bin1 fibers recorded during depolarizing pulses from −40 mV to 0 mV exhibited a sigmoidal fit showing the inward Ca^2+^ current characteristics of DHPR, a L-type voltage gated Ca^2+^ channel (Fig. 3c) [22]. Interestingly, the mean amplitude of Ca^2+^ current in the shRNA-Bin1 fibers continuously displayed reduced inward Ca^2+^ current even as early as at −30 mV depolarizing pulses. The reduction of inward Ca^2+^ current (A/F) in the shRNA-Bin1 fibers when compared to the shRNA-control was summarized as follows: at −40 mV (0.19±0.04; 0.12±0.05); at −30 mV (0.12±0.03; −0.28±0.19); at −20 mV (0.02±0.11; −0.60±0.22); at −10 mV (−1.32±0.22; −2.10±0.26); and at 0 mV (−2.61±0.31; −3.61±0.43). The altered inward Ca^2+^ current in the shRNA-Bin1 fibers could be potentially due to ineffective conformational coupling between DHPR and RyR.

In quantifying the kinetic properties of VICR, we examined two parameters: 1) amplitude (ΔF/F_0_), which defines the peak amplitude of Ca^2+^ release; and 2) the rising and corresponding decay time constant (τ) of exponential functions fit to tracings of Ca^2+^ release. Interestingly, the mean amplitude of Ca^2+^ transient evoked at different depolarizing pulses also exhibited compromised Ca^2+^ release in the shRNA-Bin1 fibers (Fig. 3d,e). The reduction of Ca^2+^ transient amplitude in the shRNA-Bin1 fibers when compared to the shRNA-control was summarized as follows: at −40 mV (0.41±0.03; 0.46±0.05); at −30 mV (0.90±0.09; 1.29±0.08); at −20 mV (1.75±0.12; 2.14±0.07); at −10 mV (2.18±0.13; 2.55±0.08); at 0 mV(2.33±0.15; 2.71±0.11). The reductions in the amplitude of both Ca^2+^ current and the shRNA- Bin1 fibers further imply that structural changes in the t-tubule membranes alter the coupling of DHPR to RyR, leading to reduced global Ca^2+^ release. This result is in accordance with a previous study showing that knockdown of Bin1 in cultured cardiomyocytes results in a delayed Ca^2+^ transient [10]. On the other hand, no significant changes were observed in the rise and decay τ of shRNA-Bin1's Ca^2+^ release when compared to the control (Fig. 3d, f), suggesting that the fundamental channel properties of RyR remain intact in the shRNA-Bin1 fibers. Additionally, the Ca^2+^ release in shRNA-control fibers was similar to untransfected wild-type fibers (data not shown), indicating that 14 days are sufficient to restore the Ca^2+^ handling properties of electroporated FDB muscle.

**Figure 4:**
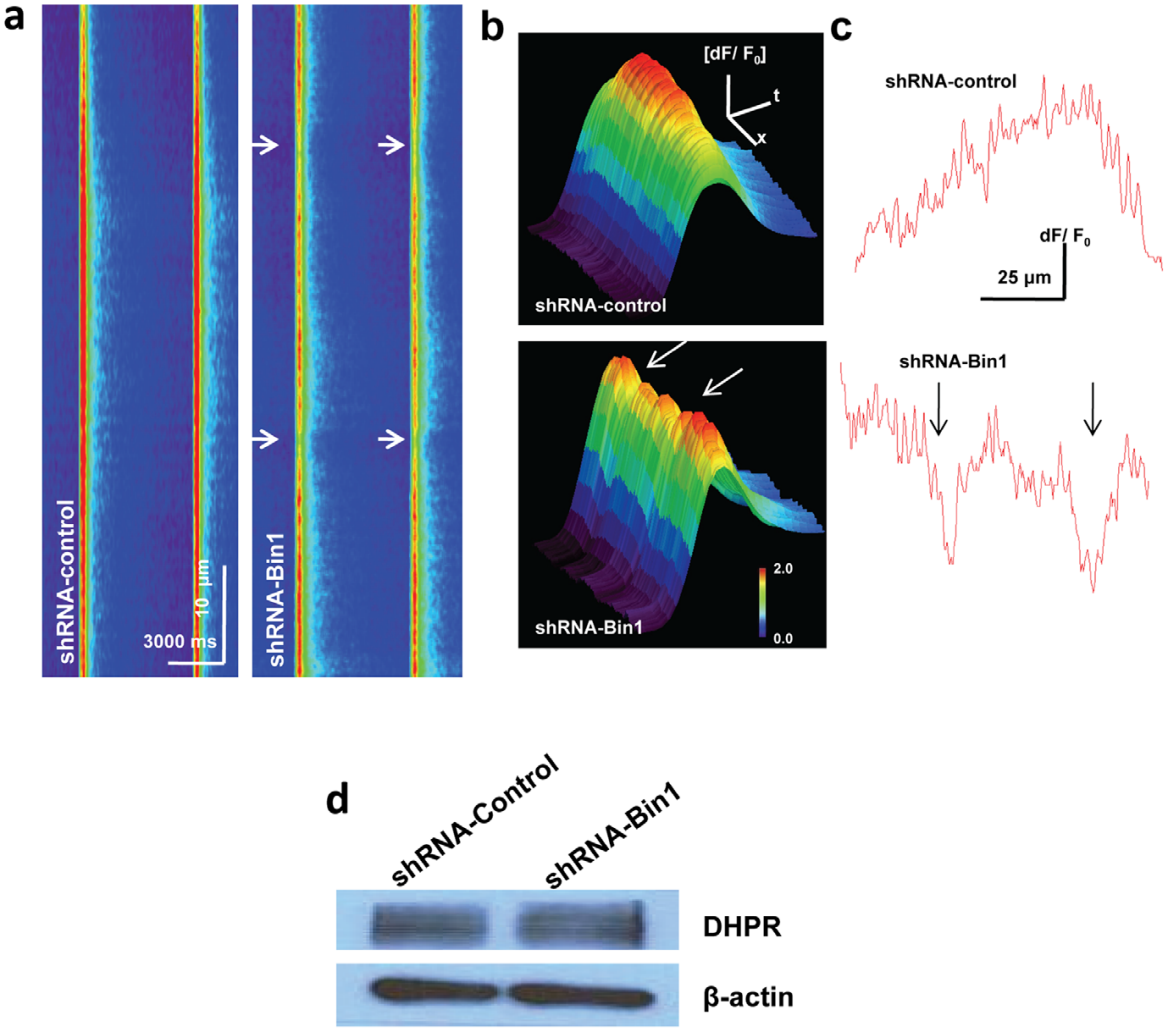
Disrupted continuity of Ca^2+^ transient in shRNA-Bin1 fibers. To establish if areas of disrupted t-tubule structure results in local alteration of the amplitude of Ca^2+^ following membrane depolarization individual fibers were incubated with 5 µM BAPTA-AM for 20 minutes before *x_t_* linescans were run on the long axis of the muscle fiber. (a) The buffering capacity of BAPTA allowed identification of altered spatial distribution of the Ca^2+^ transients (arrows) in control (left) and Bin1 knockdown (right) fibers. Images are representative Fluo-4 fluorescence measurements taken from longitudinal line scans during -20 mV depolarizing pulses for 200 ms. Similar images were collected from multiple fiber from three different wild type mice. (b) 3-D representation amplitude measurements from (a) show areas of reduced amplitude (dF/F_0_) indicating breaks in the Ca^2+^ transient of shRNA-Bin1 fibers. (c) Amplitude tracings taken from the peak of the Ca^2+^ transients in (a). In these tracings, the x axis indicates the distance across long side of the fiber while the y axis indicates the amplitude of signal at each location in the fiber. (d) Western blot showed similar expression of DHPR (Ca_V_ 1.1) in both shRNA-control and shRNA-Bin1 fibers.

As lower Ca^2+^ transient amplitude suggested compromised coupling between DHPR and RyR, we then investigated the spatial distribution of the Ca^2+^ transient in the shRNA-Bin1 fibers. Adapting the method by Yang, et al [25], we added 5 µM BAPTA-AM that will buffer Ca^2+,^ allowing us to identify breaks in the Ca^2+^ transient in the pre-incubated Fluo-4-AM fibers. With longitudinal line scan, we were able to observe discrete localized pattern of Ca^2+^ transient in the fibers that presumably corresponded to effective conformational coupling between DHPR and RyR [25]. As expected, shRNA-control fibers displayed uniform Ca^2+^ transient (Fig. 4a) when stimulated with 200 ms of −20 mV depolarizing pulses.ShRNA-Bin1 fibers (Fig. 4a), on the other hand, displayed breaks in the Ca^2+^ transient amplitude that were evidently shown by the grooves in the 3D image plot and Y plot. (Fig. 4b,c). These breaks in the Ca^2+^ transient implied compromised conformational coupling between DHPR and RyR due to disrupted t-tubule structure in the shRNA-Bin1 fibers. Longitudinal line scan of shRNA-Bin1 also showed similar reduced Ca^2+^ transient amplitude as observed in the transverse line scan (Fig. 3d,e). The expression of DHPR (Fig. 4d), however, was similar in both shRNA-control and shRNA-Bin1 fibers. These data showed that disrupted t-tubule structure in the shRNA-Bin1 fibers (Fig. 2) disrupted the conformational coupling of DHPR and RyR, as evidently shown by breaks in the Ca^2+^ transient (Fig. 4a–c) and reduced Ca^2+^ transient amplitude (Fig. 3d,e).

### Defective Ca^2+^ spark signaling in shRNA-Bin1 muscle

The reduced Ca^2+^ released upon depolarizing in the shRNA-Bin1 fibers (Fig. 3) could directly result from compromised DHPR coupling to RyR1 or from a general disruption of Ca^2+^ homeostasis due to changes in the structure of t-tubules and other intracellular membrane compartments. We tested if general disruption of intracellular Ca^2+^ homeostasis occurred using osmotic stress to produce Ca^2+^ sparks events. Previous studies have suggested that transient membrane deformation through osmotic stress depolarizes surface membrane and activates DHPR that results in increased RyR's opening probabilily (Po) generating Ca^2+^ spark events in skeletal muscle [21], [26]. As our data in Fig. 3 and 4 suggested that shRNA-Bin1 fibers exhibited altered conformational coupling between DHPR and RyR, we also tested if spontaneous resting Ca^2+^ sparks would appear more frequently in the shRNA-Bin1 fibers. As we observed in previous studies, there were only minimal spontaneous resting Ca^2+^ sparks in the electroporated shRNA- Bin1 or shRNA-contol fibers (data not shown).

**Figure 5:**
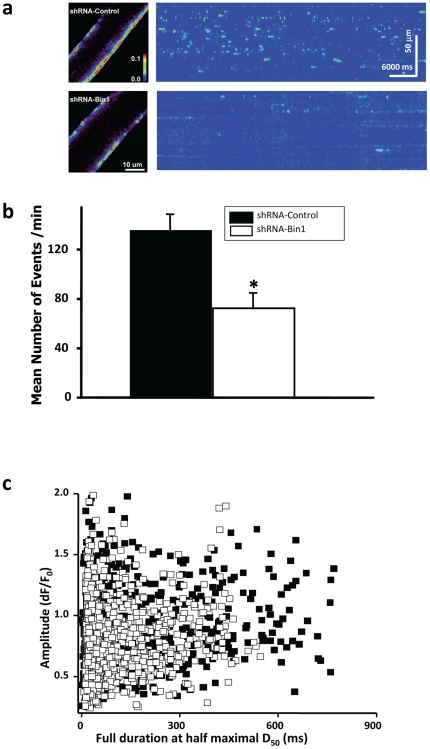
Defective Ca^2+^ spark signaling in shRNA-Bin1 muscle. Individually isolated FDB muscle fiber that expressed either shRNA-control or shRNA-Bin1 was treated with hypotonic osmotic stress to generate a Ca^2+^ sparks response. (a, *left*) Representative XY images of FLuo-4 fluorescence illustrated the sub-cellular localization of Ca^2+^ sparks. Images were pseudo-colored using an arbitrary scale with red appearing as the highest fluorescence signal (*inset*). shRNA-Bin1fibers (bottom) showed peripheral Ca^2+^ sparks localization despite reduced Ca^2+^ spark frequency when compared to shRNA-control (top). (a, *right*) Representative pseudocolored x*_t_* linescan images of either shRNA-control or shRNA-Bin1 FDB fibers following osmotic stress. The frequency and amplitude of Ca^2+^ appear to be lower in shRNA-Bin1 fibers. (b) shRNA-Bin1 (n = 12 fibers) exhibited reduced mean Ca^2+^ spark frequency per minute when compared to shRNA-control (n = 12 fibers). (c) Correlation of the amplitude (dF/F_0_) and full duration at half maximum (D_50_) of individual Ca^2+^ release events showed a population of high amplitude and long duration Ca^2+^ release events in the shRNA-control fibers (filled square) that were absent in the shRNA-Bin1 group (blank square) (n = 973 events for shRNA-control; 1128 for shRNA-Bin1).

Fibers were then subjected to osmotic stress induced Ca^2+^ sparks by perfusing hypotonic solution (osmolality = 170 mOsm) that transiently swells the muscle fiber. Rapid removal of hypotonic solution by perfusion of isotonic solution (osmolality = 290 mOsm) restored cell volume and generated robust peripheral Ca^2+^ sparks activity (Fig. 5a left) [19], [21], [26].We found that Ca^2+^ sparks signaling in shRNA-control fibers was similar to the untransfected wild-type muscle fibers (Fig. 5a). The line scan representation (x*_t_*) of the shRNA-Bin1 fibers, however, exhibit greatly reduced Ca^2+^ sparks frequency (Fig. 4a, right). The average number of Ca^2+^ sparks per minute in the shRNA-Bin1 fibers (72.46 ± 12.47) was significantly lower when compared to shRNA-control (135.6±13.0) (Fig. 5b).

In quantifying the kinetic properties of Ca^2+^ sparks, we also examined the full duration at half maximum (D_50_) which represents the time between half rising time and the corresponding decay time and amplitude (ΔF/F_0_) [27]. Correlation (Fig. 5c) of the ΔF/F_0_ and D_50_ of individual Ca^2+^ release events showed that shRNA-Bin1 Ca^2+^ spark events tend to be lower in amplitude and shorter in duration. The mean amplitude and duration of shRNA-Bin1 (0.77 ±0.008; 89.8± 3.06) were significantly lower than the shRNA-control fibers (0.85±0.01; 149.91±6.12). Additionally, shRNA-Bin1 fibers seemed to display less high amplitude and long duration Ca^2+^ events, further suggesting that knocking down Bin1 caused a general decrease in the quantity of Ca^2+^ that can be released from the SR.

### Reduced Ca^2+^ store contributes to the altered Ca^2+^release in the shRNA-Bin1 fiber

**Figure 6:**
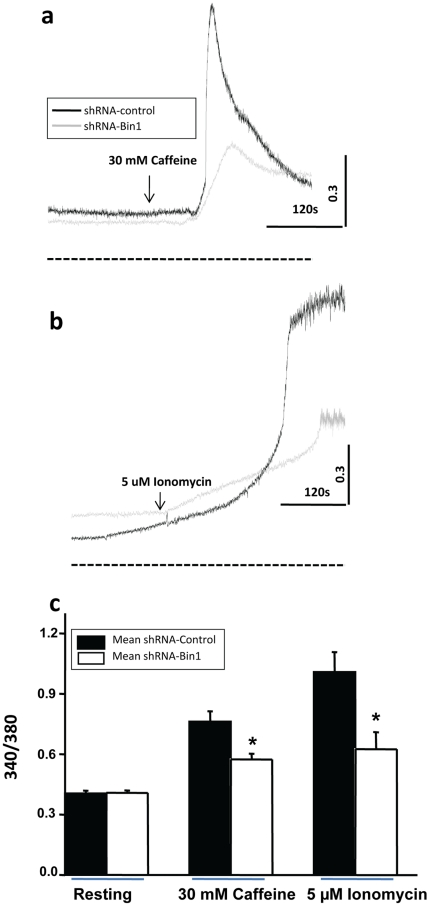
Reduced Ca^2+^ store contributes to the altered Ca^2+^ sparks in the shRNA-Bin1 fiber. Representative Fura-2 fluorescence traces of resting [Ca^2+^]_i_ and application of either (a) 30 mM Caffeine induced SR Ca^2+^ release in FDB fibers or (b) 5 µM Ionomycin- induced total [Ca^2+^]_i_ store in FDB fibers. Fiber was previously perfused with zero Ca^2+^ Tyrode before application of either caffeine or ionomycin to induce store [Ca^2+^] release. The black line represents shRNA-control fibers and the gray line represents shRNA-Bin1 fibers. The dotted line represents the level of zero fluorescence. Black dotted line in the x-axis indicates the zero values for these traces. (c) ShRNA-Bin1 fibers (blank square) (n = 20) displayed reduced mean SR and total [Ca^2+^]_i_ store when compared to shRNA-control fibers (filled square) (n = 25). Resting [Ca^2+^]_i_ levels were not affected by shRNA-Bin1.

Our results indicated that disrupted triad structure could alter Ca^2+^ release in the shRNA-Bin1 fibers. Disruption of intracellular membrane structures and concurrent changes in intracellular [Ca^2+^]_i_ storage or an altered resting [Ca^2+^]_i_ level could be potential mechanisms to explain the phenotype we observed. Measurement of resting [Ca^2+^]_i_ levels using Fura-2 ratiometric dye showed similar resting [Ca^2+^]_i_ levels in both shRNA-control and shRNA-Bin1 fibers. Application of 30 mM Caffeine, a RyR agonist, showed reduced mean SR Ca^2+^ store in the shRNA-Bin1 fibers (0.57±0.03) when compared to the shRNA-control (0.76±0.05) (Fig. 6a,c). Furthermore, application of 5 uM Ionomcyin, a Ca^2+^ ionophore, which allows release of the Ca^2+^ in all intracellular compartments of the muscle fiber [28], [29], [30], revealed a decreased mean total Ca^2+^ store in shRNA-Bin1 fibers (0.62±0.08) compared to shRNA-control fibers (1.01±0.1) (Fig. 6b,c). These findings suggest that the reduction in the SR Ca^2+^ release upon voltage depolarizing and Ca^2+^ spark amplitude in shRNA-Bin1 muscle fibers could result from reduced total [Ca^2+^]_i_ storage within some compartment of the muscle fiber rather than increased resting [Ca^2+^]_i_.

## Discussion

Studies in C2C12 cells and isolated muscle tissue showed that Bin1 is important for membrane fusion and t-tubule development [4], [5], [7]. Aberrant myofibril organization in Bin1 knockout mice resulted in cardiomyopathy and embryonic lethality, emphasizing the importance of t-tubule structure in striated muscle [11]. Recent findings have suggested that Bin1 is involved in sarcomeric protein organization in skeletal muscle and also in the maintenance of Ca^2+^ homeostasis in the heart of adult's mice [9], [10]. These findings emphasize the need to further characterize the role of Bin1 in [Ca^2+^]_i_ homeostasis in adult skeletal muscle as alteration in membrane structure could possibly affect the coupling of DHPR to RyR1, and hence [Ca^2+^] release events. Here, we show that knocking down Bin1 expression in adult FDB results in disrupted t-tubule structure (Fig. 2), suggesting that Bin1 is not only important during muscle development, but also for the maintenance of intact t-tubule structure in adult skeletal muscle.

As intact triad junction structure is critical for efficient conformational coupling between DHPR and RyR [17], it follows that shRNA-Bin1 fibers displayed breaks in Ca^2+^ transient amplitude (Fig. 4a–c) and reduced amplitude of Ca^2+^ transient upon depolarizing pulses (Fig. 3c–e).This finding was in accordance to the study performed in the cultured cardiomyocytes [10], further confirming the importance of Bin1 in maintenance of the structural components necessary to establish normal physiology Ca^2+^ signaling in striated muscle. Despite the alteration in the t-tubule structure, the channel characteristic of RyR1 was fully intact and functional, as indicated by similar rise and decay τ between shRNA-Bin1 and shRNA-control fibers. This result was expected since the SR structure was unaltered in the shRNA-Bin1 fibers as observed by the EM analysis (Fig.2).

Exercise or transient plasma membrane deformation through osmotic stress induces a robust Ca^2+^ spark response that is spatially restricted to the periphery of sarcolemmal membrane in intact mammalian muscle [20], [21], [26], [31]. Several studies have postulated that osmotic stress induces structural changes at the triad junction, mechanical deformation of the membrane, increased membranedepolarization, and DHPR activation, at which all of them could possibly contribute to increasing the open probability (Po) of RyR, hence the appearance of Ca^2+^ sparks in skeletal muscle [21], [26], [31], [32]. We observe a decrease in the frequency of Ca^2+^ sparks following osmotic shock in these fibers. Although the frequency of Ca^2+^ sparks partially depends upon the SR Ca^2+^ store [33], it is possible that the decrease in the Ca^2+^ sparks frequency in the reduced of Bin1 levels was further exacerbated with the altered DHPR function. This is supported by the IV plot obtained from our VICR experiments (Fig. 3c) showing that the functional coupling between DHPR and RyR may be altered with knockdown of Bin1 in adult skeletal muscle.

Ca^2+^ sparks are rarely observed during resting conditions in mammalian skeletal muscle fibers with intact membranes [18], [34], [35]. Considering that disruption of the inhibitory interaction between the DHPR and RyR could result on Ca^2+^ leak from the SR [16], [28], [29], one might predict that such fibers would display increased resting [Ca^2+^]_i_ levels or resting Ca^2+^ sparks. In our experiments, we observed similar resting [Ca^2+^]_i_ measurements between shRNA-Bin1 and shRNA-control fibers and no resting Ca^2+^ sparks in these fibers. This may be due to the localized effects of Bin1 knockdown as both DiOC5 staining and EM analysis showed that in the shRNA-Bin1 FDB muscle fibers, only approximately 30–40% of the t-tubule structure are disrupted (Fig. 2). The remainder 60% of intact t-tubule is sufficient to structurally and physiologically repress Ca^2+^ leak; while our shRNA- electroporation approach reduced Bin1 expression to approximately 70% of wild type levels (Fig. 1).

While a change in DHPR function appears to be an important aspect of triggering Ca^2+^ sparks following osmotic shock in skeletal muscle fibers, it does not explain the restriction of the appearance of Ca^2+^ sparks to the periphery of the muscle fiber. This suggests that there is a spatially restricted signal at the plasma membrane that contributes to the generation of Ca^2+^ sparks following osmotic stress. Reduced Bin1 expression has no apparent effect on localization of the Ca^2+^ spark response, indicating that Bin1 does not participate in the restriction of Ca^2+^ sparks to the periphery of the muscle fiber. Determination of the factors restricting Ca^2+^ spark should be one avenue of future research into this response.

In conclusion, we report that Bin1 has an important role in skeletal muscle membrane organization and [Ca^2+^]_i_ homeostasis both during development [11] and in the adult skeletal muscle.Considering that previous studies suggested a potential role of SH3 domain of Bin1 in sarcomeric protein assembly and organization in adult skeletal muscle [9], and disrupted Bin1 results in altered membrane structure and compromised [Ca^2+^] handling in cardiac muscle [10], future studies of Bin1 in skeletal muscle could address the potential role of Bin1 contractility. Human mutation of Bin1 is associated with centronuclear myopathy [36], a myopathy that also involves significant dysregulation of Ca^2+^ homoestasis in skeletal muscle. Furthermore, previous studies have determined a role of Bin1 in membrane curvature to establish the complex membrane structures seen in the t-tubules of striated muscles [7], however the molecular basis for this function has not been closely examined. Future studies could address the molecular mechanisms at work in Bin1 function in both developing and adult skeletal muscle.

## Materials and Methods

### shRNA design

Initial screening experiments for RNAi probes to suppress Bin1 containing Exon 11 were conducted using multiple short hairpin (sh)RNA oligonucleotides targeting mouse Bin1 cDNA. Plasmid containing different shRNA oligos against Bin1 was then co-transfected with HA- tagged murine Bin1 cDNA to HEK293 cells to test the probe efficacy. 96 hours post-transfection, the cells were collected and prepared for western blot analysis. These results indicated that shRNA against exon 8 was the most effective in silencing endogenous expression of Bin1 containing exon 11. The shRNA oligonucleotides against exon 8 of Bin1 (shRNA-Bin1):5′GATCCGCGTGTAGGTTTCTATG TCAACATTCAAGAGATGTTGACATAGAAACCTACACGCTTTTTTG3′ and 5′AATTCAAAAAA GCGTGTAGGTTTCTATGTCAACATCTCTTGAATGTTGGACATAGAAACCTACACGCG3′ or, as a control, shRNA targeting a sequence in the luciferase cDNA (shRNA-control): 5′GATCCGTGCGCTGCTGGTGCCAACTTCAAGAGATTTTTTGCTAGCG3′, and 5′AATTCGCTAGCAAAAAATCTCTTGAAGTTGGTACTAGCAACGCACG3′, were synthesized [17]. These oligonucleotides were annealed to the *Bam*HI and *Eco*RI restriction sites of the pSIREN-DNR plasmid (Clontech, Palo Alto, CA) to generate shRNA interference probes. A separate plasmid pU6-mRFP was derived from the pShuttle vector (Clontech) by two subcloning steps. First, a red fluorescent protein (RFP) cDNA was inserted behind the cytomegalovirus promoter. Second, the U6-shRNA cassette from pSIREN-DNR was ligated into the pShuttle backbone, resulting in the generation of pU6-mRFP. This plasmid forms the backbone of the shRNA-Bin1 and shRNA-control constructs used in these experiments.

### Electroporation of plasmid DNA into adult muscle

3–6 month old male wild-type mice from C57Bl/J6 background were anesthetized with ketamine (90 mg/kg) and xylazine (10 mg/kg) by intraperitoneal injection before injection of 15 uL of 2 mg/mL hyaluronidase (Sigma #H4272) into the footpad of mouse. 60 minutes later, 20 uL (2 ug/uL) of endotoxin-free plasmid DNA of shRNA-Bin1 or shRNA-control in 0.9% saline is injected into the area of the foot surrounding FDB muscle. After 15 min to allow diffusion of the plasmid, two 13 mm steel acupuncture needles were inserted on either side of the FDB, perpendicular to the muscle. Twenty pulses of a 100 V/cm electric field were applied in 20 ms bursts with a burst interval of 100 ms between the two needles. Mice were returned to their cages and monitored every half hour until they recovered from the anesthetics. Mice that showed any sign of inflammation on their electroporated feet were excluded from all of the experiments mentioned. For all experimental results reported here, mice were sacrificed at 14 days post electroporation and FDB muscles were surgically removed. Killing of the animals and tissue preparation were conducted using protocols approved by the UMDNJ-RWJMS IACUC (protocol number I10-028-3).

RFP expression was used as an indicator to measure the transfection efficiency. In the sample preparation for western blot analysis, a portion of the muscle bundle in the transfected FDB muscle bundle that did not express RFP expression was trimmed out from the remaining muscle mass and excluded. The transfected muscle bundle was then homogenized as previously described [27] and the resulting protein lysate was run into an 8% SDS-PAGE gel. The protein was transferred into an Immobilon PVDF membrane (Millipore, Bedford, MA) and probed with monoclonal antibody against mouse Bin1 (Santa Cruz, Ca) (1∶1000) or DHPR (Ca_V_ 1.1) (ABR) (1∶1000).

### Enzymatic dissociation of individual FDB fibers

FDB muscles transfected with either shRNA-control or shRNA-Bin1 were surgically removed in 0 Ca^2+^ Tyrode buffer, (in mM) 140 NaCl, 5 KCl, 10 HEPES, 2 MgCl_2_ (pH 7.2), and incubated in Tyrode buffer containing 2 mg/mL type I collagenase (Sigma C-5138) for 90 minutes at 37°C. After two washes in isotonic Tyrode buffer containing 2.5 mM Ca^2+^ (osmolality = 290 mOsm), muscle fibers were gently dissociated by several passages through a series of micropipetter tips of gradually decreasing diameter. Individual FDB muscle fibers were plated onto ΔTC3 glass-bottomed Petri dishes (Fisher Scientific) in Tyrode and used for experimentation within 6 hours.

### DiOC5 t-tubule staining

Individual FDB fibers were loaded with 100 nM DiOC5, a membrane impermeable dye, for 20 minutes at room temperature. After washing, red transfected fibers with intact sarcolemmal membranes and regular striation patterns were selected by epi-fluorescence microscopy. T-tubule staining was observed on a BioRad Radiance-2100 confocal microscope equipped with Argon laser (479 nm), Helium-Neon laser (632 nm), and a 40X, 1.3NA oil immersion objective. X-y images were acquired at 20 s per frame (512×512 pixels). Using ImageJ software, several regions of interests (ROI) were drawn on the areas of the fibers that displayed blank DiOC5 staining (numerically labeled small ROIs) and then the total affected area in each fiber was divided by the total surface area of the fiber (large ROI drawn covering the surface area of the fiber).

### Electron Microscopy

Freshly dissected electroporated FDB muscle bundle was fixed in 2.5% glutaraldehyde and 0.1 M cacodylate buffer (pH 7.4) and later post fixed in 1% OsO_4_ and 0.1 M cacodylate buffer (pH 7.4). Samples were processed and examined as described previously [17].

### Voltage induced Ca ^2+^ release measurement using silicone grease embedding

This approach was adapted from the methods developed by Jacquemond and colleagues [22], [23]. Individually isolated FDB muscle fibers were plated onto a silicone coated dish that contains DMEM + 10% FBS. The dish was loaded with Fluo4-AM (10 µM) and incubated for 45 minutes at 37°C in a 5% CO_2_ incubator. Dish containing fibers was then washed three times by replacing the media with TEA containing solution as extracellular medium (in mM, pH  = 7.4) (140 TEA-CH3SO3, 2.5 CaCl2, 2 MgCl2, 10 HEPES, 0.001 Tetrodotoxin, and freshly-added 1 4-aminopyridine, 0.1 anthracene-9-carboxylic acid). 20 µM N-benzyl-p-toluene sulphonamide (BTS) was applied for 15 min to prevent motion artifact from muscle contraction. Fibers selected for analysis were confirmed to express RFP, in addition to having intact sarcolemmal membranes and regular striation patterns observable by phase contrast microscopy. Fiber was then silicone coated on both sides, leaving a small surface area exposed to the extracellular solution (Fig. 3a). An Axon 200 Patchlamp amplifier (Axon instruments) was used in whole cell configuration. Ca^2+^ imaging during command voltage pulse generation was recorded using BioRad Radiance-2100 confocal microscope equipped with an argon laser (488 nm) and a 40x, 1.3 NA oil-immersion objective. Resulting x*_t_* line scans of Furo-4 fluorescence were analyzed and fit to exponential functions using custom IDL software routines that were previously described [19], [21], [24], [26]. Analog compensation was systematically used to decrease the effective series resistance. Voltage-clamp was performed with a microelectrode filled with internal like solution (in mM, pH  = 7.4) (140 K-glutamate, 5.5 MgCl2, 5 HEPES, 5 glucose, 1 EGTA) Microelectrode resistance was within 0.5 MΩ–0.9 MΩ. Fiber was impaled by inserting the tip of the microelectrode through the silicone, within the insulated part of the fiber. The mean measured capacitance was 319±24.95 pF for shRNA-Bin1 and 447±35.26 pF for shRNA-control fibers. The mean input resistance calculated from the steady-state change in membrane current evoked by 10 mV hyperpolarizing command step of 20 ms duration was 7.15±0.64 MΩ and 6.36±0.45 MΩ for shRNA-Bin1 and shRNA-control, respectively. Measurement of capacitance and membrane surface area was taken for each fiber following the detailed protocol from Jacquemond and colleagues [22], [23] (Fig. 3a). Surface area was calculated from the measured length and width of the free portion of fiber, assuming it to be a cylinder. In experiments where longitudinal line scans were used to measure the spatial distribution of the Ca^2+^ transient amplitude, 5 µM BAPTA-AM was added to the muscle fibers 20 minutes before the end of the loading period for Fluo-4 AM.

### Ca^2+^ spark analysis

After confirming the RFP expression, individually isolated FDB muscle fibers were loaded with Fluo4-AM (10 µM) for 60 min at room temperature. Measurements of Ca^2+^ sparks were performed using a BioRad Radiance-2100 confocal microscope equipped with an argon laser (488 nm) and a x40, 1.3 NA oil-immersion objective. For Ca^2+^ spark measurements, fibers were perfused with hypotonic solution (osmolality  = 170 mOsm) containing (in mM) 70 NaCl, 5 KCl, 10 Hepes, 2.5 CaCl_2_, 2 MgCl_2_ (pH 7.2), for 100 seconds to induce swelling before perfusion was switched back to the initial isotonic Tyrode buffer (osmolality  = 290 mOsm). The osmolality of all solutions were measured using a Micro Osmometer 3300 (Advanced Instruments). Line scan images of 512 pixels in length were acquired at a sampling rate of 2 ms per line, and serial x-y images of muscle fibers were acquired at 3.08 s per frame. Image analysis of Fluo-4 fluorescence in both VICR and Ca^2+^ spark experiments was performed using IDL software and customer devised routines [19], [21], [24], [27].

### Resting and store Ca^2+^ measurement

After confirming the RFP expression, individually isolated FDB muscle fibers were loaded with 10 µM fura-2 AM for 45 min at room temperature in isotonic Tyrode solution. 20 µM BTS was applied for 15 min to prevent motion artifact from muscle contraction. The ratio of fura-2 fluorescence at excitation wavelength of 340 and 380 nm was measured on a PTI spectrofluorometer (Photon Technology International) to assess the resting [Ca^2+^]_i_ level. Zero Ca^2+^ Tyrode solution was perfused to the fiber before either adding 30 mM Caffeine or 5 µM ionomycin (Sigma) to induce [Ca^2+^] store released.

### Statistical analysis

Results are present as mean ± SEM as tested for statistical significance by t-test, *p<0.05. Statistical analysis was performed using Origin software. In each experimental group, fibers were isolated from 8 mice, and from each mouse n≥3 fibers were used for analysis.

## References

[1] Ren G, Vajjhala P, Lee JS, Winsor B, Munn AL (2006). The BAR domain proteins: molding membranes in fission, fusion, and phagy.. Microbiol Mol Biol Rev.

[2] Peter BJ, Kent HM, Mills IG, Vallis Y, Butler PJ (2004). BAR domains as sensors of membrane curvature: the amphiphysin BAR structure.. Science.

[3] Sakamuro D, Elliott KJ, Wechsler-Reya R, Prendergast GC (1996). BIN1 is a novel MYC-interacting protein with features of a tumour suppressor.. Nat Genet.

[4] Wechsler-Reya RJ, Elliott KJ, Prendergast GC (1998). A role for the putative tumor suppressor Bin1 in muscle cell differentiation.. Mol Cell Biol.

[5] Butler MH, David C, Ochoa GC, Freyberg Z, Daniell L (1997). Amphiphysin II (SH3P9; BIN1), a member of the amphiphysin/Rvs family, is concentrated in the cortical cytomatrix of axon initial segments and nodes of ranvier in brain and around T tubules in skeletal muscle.. J Cell Biol.

[6] Wechsler-Reya R, Sakamuro D, Zhang J, Duhadaway J, Prendergast GC (1997). Structural analysis of the human BIN1 gene. Evidence for tissue-specific transcriptional regulation and alternate RNA splicing.. J Biol Chem.

[7] Lee E, Marcucci M, Daniell L, Pypaert M, Weisz OA (2002). Amphiphysin 2 (Bin1) and T-tubule biogenesis in muscle.. Science.

[8] Razzaq A, Robinson IM, McMahon HT, Skepper JN, Su Y (2001). Amphiphysin is necessary for organization of the excitation-contraction coupling machinery of muscles, but not for synaptic vesicle endocytosis in Drosophila.. Genes Dev.

[9] Fernando P, Sandoz JS, Ding W, de Repentigny Y, Brunette S (2009). Bin1 SRC homology 3 domain acts as a scaffold for myofiber sarcomere assembly.. J Biol Chem.

[10] Hong TT, Smyth JW, Gao D, Chu KY, Vogan JM (2010). BIN1 localizes the L-type calcium channel to cardiac T-tubules.. PLoS Biol.

[11] Muller AJ, Baker JF, DuHadaway JB, Ge K, Farmer G (2003). Targeted disruption of the murine Bin1/Amphiphysin II gene does not disable endocytosis but results in embryonic cardiomyopathy with aberrant myofibril formation.. Mol Cell Biol.

[12] DiFranco M, Quinonez M, Capote J, Vergara J (2009). DNA transfection of mammalian skeletal muscles using in vivo electroporation..

[13] Jimenez-Moreno R, Wang ZM, Messi ML, Delbono O (2010). Sarcoplasmic reticulum Ca(2+) depletion in adult skeletal muscle fibres measured with the biosensor D1ER..

[14] Pouvreau S, Royer L, Yi J, Brum G, Meissner G (2007). Ca(2+) sparks operated by membrane depolarization require isoform 3 ryanodine receptor channels in skeletal muscle.. Proc Natl Acad Sci U S A.

[15] Cai C, Masumiya H, Weisleder N, Matsuda N, Nishi M (2009). MG53 nucleates assembly of cell membrane repair machinery.. Nat Cell Biol.

[16] Zhou J, Yi J, Fu R, Liu E, Siddique T (2010). Hyperactive intracellular calcium signaling associated with localized mitochondrial defects in skeletal muscle of an animal model of amyotrophic lateral sclerosis.. J Biol Chem.

[17] Hirata Y, Brotto M, Weisleder N, Chu Y, Lin P (2006). Uncoupling store-operated Ca2+ entry and altered Ca2+ release from sarcoplasmic reticulum through silencing of junctophilin genes.. Biophys J.

[18] Lee EH, Lopez JR, Li J, Protasi F, Pessah IN (2004). Conformational coupling of DHPR and RyR1 in skeletal myotubes is influenced by long-range allosterism: evidence for a negative regulatory module.. Am J Physiol Cell Physiol.

[19] Weisleder N, Brotto M, Komazaki S, Pan Z, Zhao X (2006). Muscle aging is associated with compromised Ca2+ spark signaling and segregated intracellular Ca2+ release.. J Cell Biol.

[20] Weisleder N, Ma JJ (2006). Ca2+ sparks as a plastic signal for skeletal muscle health, aging, and dystrophy.. Acta Pharmacol Sin.

[21] Wang X, Weisleder N, Collet C, Zhou J, Chu Y (2005). Uncontrolled calcium sparks act as a dystrophic signal for mammalian skeletal muscle.. Nat Cell Biol.

[22] Collet C, Csernoch L, Jacquemond V (2003). Intramembrane charge movement and L-type calcium current in skeletal muscle fibers isolated from control and mdx mice.. Biophys J.

[23] Jacquemond V (1997). Indo-1 fluorescence signals elicited by membrane depolarization in enzymatically isolated mouse skeletal muscle fibers.. Biophys J.

[24] Yazawa M, Ferrante C, Feng J, Mio K, Ogura T (2007). TRIC channels are essential for Ca2+ handling in intracellular stores.. Nature.

[25] Yang D, Pan Z, Takeshima H, Wu C, Nagaraj RY (2001). RyR3 amplifies RyR1-mediated Ca(2+)-induced Ca(2+) release in neonatal mammalian skeletal muscle.. J Biol Chem.

[26] Pickering JD, White E, Duke AM, Steele DS (2009). DHPR activation underlies SR Ca2+ release induced by osmotic stress in isolated rat skeletal muscle fibers.. J Gen Physiol.

[27] Weisleder N, Ferrante C, Hirata Y, Collet C, Chu Y (2007). Systemic ablation of RyR3 alters Ca2+ spark signaling in adult skeletal muscle.. Cell Calcium.

[28] Toth A, Ivanics T, Ruttner Z, Slaaf DW, Reneman RS (1998). Quantitative assessment of [Ca2+]i levels in rat skeletal muscle in vivo.. Am J Physiol.

[29] Brotto MA, Nagaraj RY, Brotto LS, Takeshima H, Ma JJ (2004). Defective maintenance of intracellular Ca2+ homeostasis is linked to increased muscle fatigability in the MG29 null mice.. Cell Res.

[30] Glund S, Treebak JT, Long YC, Barres R, Viollet B (2009). Role of adenosine 5′-monophosphate-activated protein kinase in interleukin-6 release from isolated mouse skeletal muscle.. Endocrinology.

[31] Martins AS, Shkryl VM, Nowycky MC, Shirokova N (2008). Reactive oxygen species contribute to Ca2+ signals produced by osmotic stress in mouse skeletal muscle fibres.. J Physiol.

[32] Teichmann MD, Wegner FV, Fink RH, Chamberlain JS, Launikonis BS (2008). Inhibitory control over Ca(2+) sparks via mechanosensitive channels is disrupted in dystrophin deficient muscle but restored by mini-dystrophin expression.. PLoS ONE.

[33] Launikonis BS, Zhou J, Santiago D, Brum G, Rios E (2006). The changes in Ca2+ sparks associated with measured modifications of intra-store Ca2+ concentration in skeletal muscle.. J Gen Physiol.

[34] Shirokova N, Garcia J, Rios E (1998). Local calcium release in mammalian skeletal muscle.. J Physiol.

[35] Murayama T, Oba T, Kobayashi S, Ikemoto N, Ogawa Y (2005). Postulated role of interdomain interactions within the type 1 ryanodine receptor in the low gain of Ca2+-induced Ca2+ release activity of mammalian skeletal muscle sarcoplasmic reticulum.. Am J Physiol Cell Physiol.

[36] Toussaint A, Cowling BS, Hnia K, Mohr M, Oldfors A (2010). Defects in amphiphysin 2 (BIN1) and triads in several forms of centronuclear myopathies..

